# Infrared Machine Vision System Based on Te NWs‐Au NPs Plasmonic Optoelectronic Memristor for Motion Detection

**DOI:** 10.1002/advs.202523162

**Published:** 2026-01-30

**Authors:** Jingyao Bian, Yongxing Zhu, Ye Tao, Zhongqiang Wang, Xiaoning Zhao, Ya Lin, Haiyang Xu, Yichun Liu

**Affiliations:** ^1^ State Key Laboratory of Integrated Optoelectronics Ministry of Education Key Laboratory for UV Light‐Emitting Materials and Technology (Northeast Normal University) Changchun P. R. China; ^2^ School of Science Heilongjiang University of Science and Technology Harbin P. R. China

**Keywords:** Au nanoparticles, infrared vision system, localized surface plasmon resonance, optoelectronic memristor, Te nanowires

## Abstract

Infrared (IR) machine vision systems have advanced significantly in a range of applications, including autonomous driving, security monitoring, and intelligent night vision. Despite advances in optoelectronic memristors by mixed optical and electrical operation, achieving infrared‐specific fully light‐modulated vision systems remains challenging. Here, we demonstrate a plasmonic optoelectronic memristor based on Te nanowires‐Au nanoparticles/ι‐carrageenan film. Localized surface plasmon resonanceassisted optical excitation endows the device with non‐volatile, IR‐programmable conductance states that can be selectively erased by visible light, yielding an all‐photonic write/erase scheme without electrical intervention. Exploiting this reversible photonic plasticity, we construct an IR vision system capable of in‐sensor Boolean logic and motion detection under complete darkness. An optical neural network trained on the reversible conductance dynamics attains 91.4% recognition accuracy for moving objects. This work proposes a fully light‐modulated optoelectronic memristor that may promote the future development of efficient IR machine vision systems.

## Graphical abstract

A plasmonic optoelectronic memristor based on Te nanowires‐Au nanoparticles/ι‐carrageenan enables IR‐programmed and visiblelight‐erased non‐volatile conductance. The all‐photonic write/erase scheme supports in‐sensor logic and real‐time motion detection in darkness. An artificial neural network trained on reversible conductance achieves 91.4% recognition accuracy, advancing efficient IR machine vision systems.

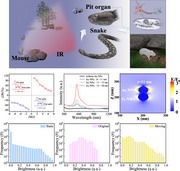

## 1 Introduction

Infrared (IR) machine vision system has garnered significant attention for real time image recognition and motion detection in low‐light conditions, owing to their capability to efficiently perceive, convert, and process vast amounts of IR optical information required in contemporary applications [1, 2, 3]. Conventional IR vision systems built on the von Neumann architecture are plagued by substantial redundant data generation and high energy consumption, largely resulting from the physical separation between sensing and processing modules [4]. For instance, Lee's team integrated a transistor and a photodetector to implement IR optical‐sensing and memory functions for achieving the optical sensory system [5]. Wang et al. reported a visual nervous system combining IR sensors and memristors to demonstrate a dynamic gesture perception task [6]. These efforts have not only enhanced the sensing and memory performance of the IR machine vision system but have also advanced neuromorphic device designs that represent state‐of‐the‐art research.

Optoelectronic memristors that merge IR sensing with in‐memory processing present a promising platform for machine vision applications under dark environments [7, 8, 9]. 1D/2D materials, featuring broadband spectral detection, atomically thin geometries, and narrow bandgaps, have emerged as attractive building blocks for such optoelectronic memristors [10, 11, 12, 13]. Notably, IR light‐induced synaptic potentiation and depression have been demonstrated in several 1D/2D material systems. For instance, Ye et al. reported a TiS_3_‐based optoelectronic artificial synapse in which IR photoinduced carriers facilitate the formation of conductive filaments (CFs) and the subsequent electrical rupture of these CFs [12]. Dong et al. presented a graphene‐based derivative photonic synapse, showing persistent IR photoconductivity effect and inhibitory electrical behavior [13]. These results underscore the importance of coupled optical and electrical stimuli in triggering reconfigurable excitatory and inhibitory synaptic responses, thereby advancing IR vision systems. All‐optical modulation optoelectronic memristors for IR sensing thus constitute a highly promising direction for the next‐generation IR machine vision.

The localized surface plasmon resonance (LSPR) in Au or Ag nanoparticles enables spectrally tunable photoresponses from the visible to IR range, which is critical for bidirectional synaptic modulation [14, 15, 16, 17, 18]. Recently, Li et al. reported a broadband optoelectronic memristor enabled by Ag nanoparticles and oxygen vacancies, achieving enhanced plasmonic effects, low‐power operation, and effective synaptic emulation for real‐time intrusion detection, laying a solid foundation for the development of high‐density storage and intelligent hardware‐based security solutions [16]. In our prior work, we reported a plasmonic optoelectronic memristor based on Ag‐TiO_2_ nanocomposites, which utilizes localized surface plasmon resonance and optical excitation to achieve fully light‐modulated synaptic plasticity [9]. Building on this foundation, the Te nanowires (Te NWs)‐Au nanoparticles (Au NPs) nanocomposite offers an excellent platform for constructing an LSPR‐driven optoelectronic memristor with full optical control. However, such systems remain largely unexplored. In this work, we demonstrate a plasmonic optoelectronic memristor based on the Au/Te NWs‐ Au NPs/ι‐carrageenan (ι‐car)/Au planar structure. By leveraging the narrow bandgap of Te and the plasmon resonance of Au NPs, we achieve fully light‐induced synaptic plasticity within a single device. Thanks to polymer molecular entanglement and intrinsic flexibility, the optoelectronic memristor provides an optical pathway to flexible synapses for wearable applications. In addition, the device exhibits reversible optical switching behavior, facilitating logic gate operations. Significantly, our memristor allows for the detection of static and moving objects via interframe differential computation. Subsequently, the extracted motion information is accurately classified using an artificial neural network (ANN), achieving an accuracy of 91.4%. This work proposed here provides a feasible approach toward the construction of a highly efficient IR vision system.

## 2 Results and Discussion

### 2.1 Optoelectronic Memristor‐Based IR Vision System

**FIGURE 1:**
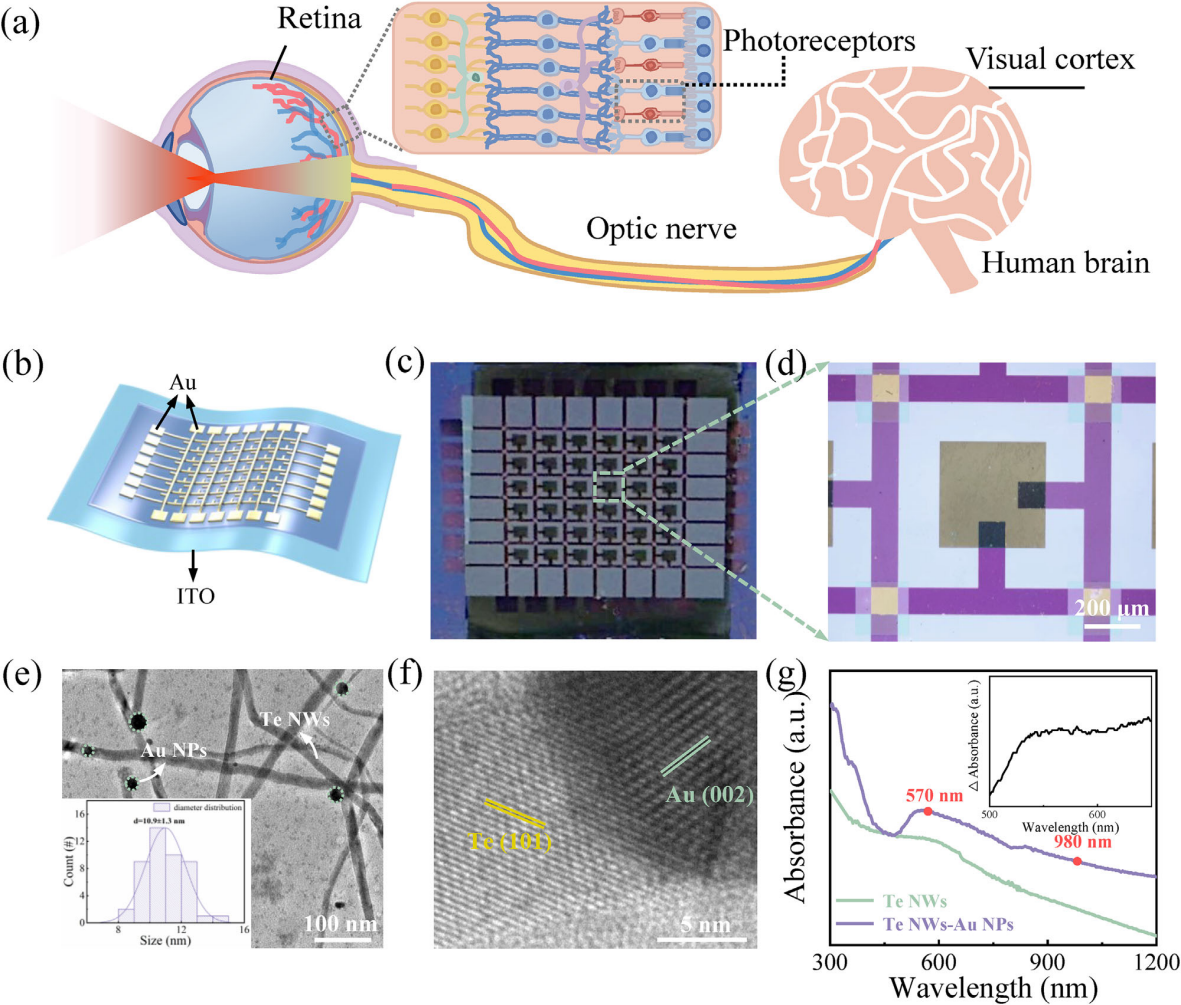
Plasmonic optoelectronic memristor‐based IR vision systems. (a) Schematic diagrams of the human visual system. (b) Schematic illustration of the planar device. (c, d) The photograph and optical image of the optoelectronic memristor array. The array consists of 6 × 6 optoelectronic memristors. (e) TEM image of the Te NWs‐Au NPs film. Inset: statistical size distribution of the Au NPs. (f) HRTEM image of the Te NWs‐Au NPs film. (g) Absorption spectra of the Te NWs‐Au NPs film and the pure Te NWs film.

Figure 1a schematically illustrates the human visual system, in which image information from the external environment is captured by the eye. Light‐carrying visual data is focused onto the retina, where photoreceptor cells convert light signals into electrical signals via photoelectric conversion [19]. The integrated information is subsequently transmitted to the optic nerve cells and ultimately to the brain. Moreover, bidirectional signal transmission supports dynamic image memory formation and erasure during behavioral learning, enabling continuous updating and recognition of visual information [20].

Inspired by the biological visual pathway, we developed a plasmonic optoelectronic memristor that integrates both visual sensing and image processing functionalities. Figure 1b presents a schematic illustration of the proposed plasmonic optoelectronic memristor, which consists of an Au/Te NWs‐Au NPs/ι‐car/Au planar structure (see Section 4 for details). The ι‐car polymer serves as a flexible and robust matrix that endows the functional layer with mechanical flexibility and structural integrity—properties essential for developing flexible electronic devices—while the corresponding Fourier transform infrared spectroscopy and molecular structure are provided in Figure S1 [21]. Moreover, the Te NWs‐Au NPs composite material can be easily dispersed in ι‐car due to molecular entanglement. A cross‐sectional scanning electron microscope image (Figure S2) reveals that the optoelectronic functional layer has a uniform thickness of approximately 480 nm. The optical view of the fabricated 6 × 6 memristor crossbar array is depicted in Figure 1c,d, in which the electrode size can be determined as 100 µm according to the optical image. Notably, the Te NWs‐Au NPs memristors exhibit excellent cycle‐to‐cycle and device‐to‐device uniformity (see Figures S3 and S4), serving as a stable foundation for the implementation of biological functions in subsequent studies.

Figure 1e presents a magnified transmission electron microscope (TEM) image, showing Au NPs densely decorated on Te NWs, with an average diameter of ∼ 11 nm (size distribution shown in the inset). High‐resolution transmission electron microscope (HRTEM) imaging in Figure 1f confirms the crystalline structure of both components, with discernible lattice fringes corresponding to the (002) plane of Au and the (101) plane of Te NWs [18]. The composite structure is further verified by energy‐dispersive X‐ray spectroscopy (EDX) and elemental mapping (Figure S5). Figure 1g shows the absorption spectra of the pure Te NWs film and the Te NWs‐Au NPs nanocomposite film. The Te NWs film exhibits broadband absorption ranging from 350 to 1200 nm, attributed to the narrow bandgap of Te. In contrast, the Au NPs‐Te NWs film demonstrates enhanced absorption in the visible region (400–800 nm), which can be ascribed to the LSPR effect of the Au NPs, in agreement with prior studies [22, 23]. Based on these optical properties, light at 980 nm (IR light) and 570 nm (visible light) was selected for subsequent optoelectronic measurements.

### 2.2 Fully Light‐Modulated Controlled Synaptic Plasticity

**FIGURE 2:**
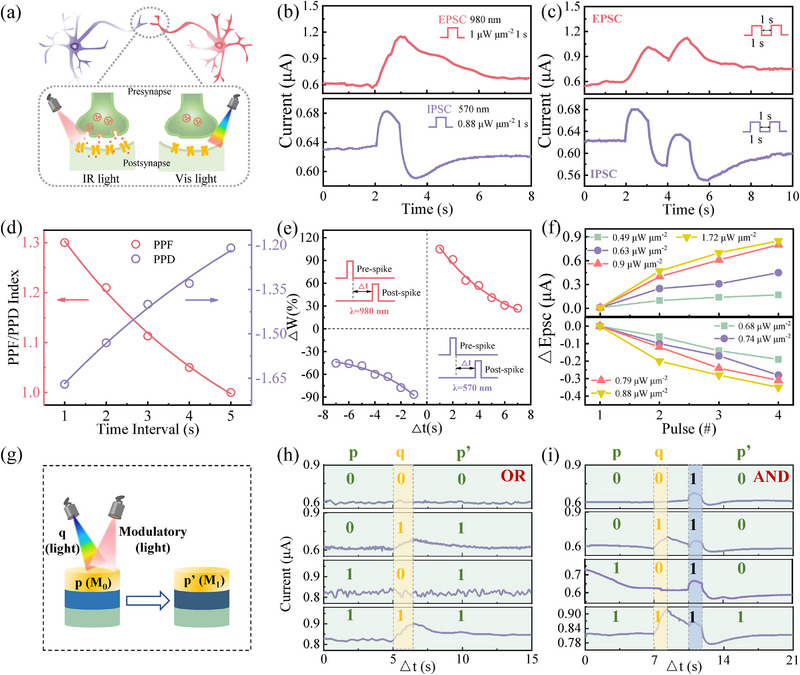
Bidirectional synaptic behavior and logic operation of optoelectronic memristor. (a) Schematic illustration of the light‐modulated synaptic weights under IR (980 nm) and visible (570 nm) light stimuli. (b, c) EPSC and IPSC responses triggered by a single and double optical pulse under 980 and 570 nm illumination, respectively, with a read voltage of 20 mV. (d) PPF (PPD) index curves as a function of the interval between optical pulses. (e) Dependence of the STDP‐induced ΔW on the interval time between the presynaptic and postsynaptic spikes (Δt_pre‐post_). (f) Light‐induced EPSC and IPSC are functions of the optical pulse numbers with various intensities. (g–i) Fully light‐modulated logic operations in the plasmonic optoelectronic memristor.

To characterize the bidirectional photoresponse of the optoelectronic memristor, we systematically evaluated its performance under optical stimulation. As illustrated in Figure 2a, the device was programmed with a constant reading voltage of 20 mV, and its current responses were recorded under illumination with both 980 and 570 nm light. As shown in Figure 2b, excitatory postsynaptic current (EPSC) and inhibitory postsynaptic current (IPSC) behaviors were triggered by applying optical pulses at 980 nm (1 µW µm^−2^, 1 s) and 570 nm (0.88 µW µm^−2^, 1 s), respectively. Under IR illumination, the current increased transiently and then gradually decayed to an intermediate state, reflecting long‐term potentiation (LTP) behavior [24]. In contrast, illumination with 570 nm light induced an eventual current decrease, signifying long‐term depression (LTD) (Figure 2b). Notably, upon visible light exposure, the current initially rose abruptly before decaying to a level below the baseline, a response analogous to IPSC. This phenomenon may be attributed to the p‐type Te NWs and LSPR effect under 570 nm light illumination, which will be elaborated later in the operating mechanism section. These results confirm that the synaptic behavior of both LTP and LTD can occur reversibly at the same synapse device under stimulation by 980 and 570 nm light, enabling full light‐controlled bidirectional plasticity.

Furthermore, temporal correlation in the optoelectronic memristor was examined using the non‐overlapping spikes. As shown in Figure 2c, the paired pulse facilitation (PPF) behavior was emulated by applying two successive optical spikes with varying time intervals (Δt) to the device (Note S1) [25]. Figure 2d illustrates that the PPF (or paired pulse depression, PPD) index exhibits a double‐exponential decay index from 1.3 (‐1.22) to 1 (‐1.65) as Δt increases from 1 to 7 s, closely resembling the behavior in biological synapses. The decay curves were well fitted using a double‐exponential equation, consistent with previously reported models [26, 27]. The spike timing dependent plasticity (STDP) governs the synaptic weight change (ΔW) depending on the timing between presynaptic and postsynaptic spikes and is regarded as a bidirectional learning rule in ANN [28]. As shown in Figure 2e, ΔW was experimentally extracted and numerically fitted by applying paired optical pulses to the Au electrodes of the device. Herein, ΔW is defined as ΔW = (G_2_−G_1_)/G_1_, where G_1_ and G_2_ are the long‐term conductance values measured before and after pulse stimulation. If the presynaptic spike occurs before the postsynaptic spike, the synapse weight is enhanced, e.g., LTP, and vice versa. The ΔW‐Δt relationship can be calculated with the following exponential function [25, 26, 27]:

$$\Delta W=A_{+}e^{\Delta t/\tau_{+}}\;\;\Delta \;t>0$$

$$=A_{-}e^{\Delta t/\tau_{-}}\;\;\Delta \;t<0$$

here, A_+_ and A_−_ represent the maximum synaptic weight increase and decrease relative to the initial state, respectively. τ_+_ and τ_−_ are time constants that determine how rapidly synaptic weights update. These temporal correlations can provide the dynamic basis to demonstrate the spatio‐temporal information processing. The above results suggest that our memristor combines two key properties, namely image sensing and processing.

Boolean logic is a two‐valued algebraic system in which variables take on binary truth values, typically represented as “1” (true) and “0” (false) [29, 30, 31, 32]. Leveraging the reversible light‐modulated characteristics, the reconfigurable logic function of OR and AND operations can be performed as logic‐in‐memory computing architectures to memory and process information. In this system, the logical inputs are encoded by light stimuli. The primary input p corresponds to the device's initial current state (M_0_), while input q and modulatory light are introduced using either 980 or 570 nm light. The logic output p’ is read as the final current state M_1_ under a constant reading voltage (+20 mV), applied synchronously with the optical input (Figure 2g). For the OR logical operation, a current threshold of 0.62 µA is defined as the baseline to distinguish logical levels: p = 0 if M_0_< 0.62 µA, and p = 1 if M_0_ ≥ 0.62 µA. The final current M_1_ is similarly interpreted as logic “1” when the enhanced current exceeds this threshold, either due to a high initial state or the presence of modulating light (Figure 2h). For the AND logical operation, the final current M_1_ is similarly interpreted as logic “1” when the enhanced current exceeds this threshold, either due to a high initial state or the presence of modulating light, as shown in Figure 2i. Thus, such optoelectronic memristors possess the feasibility of optical sensing, processing, and logical computing, providing a promising platform for neuromorphic IR vision systems.

### 2.3 Operation Mechanism of Optoelectronic Memristor

**FIGURE 3:**
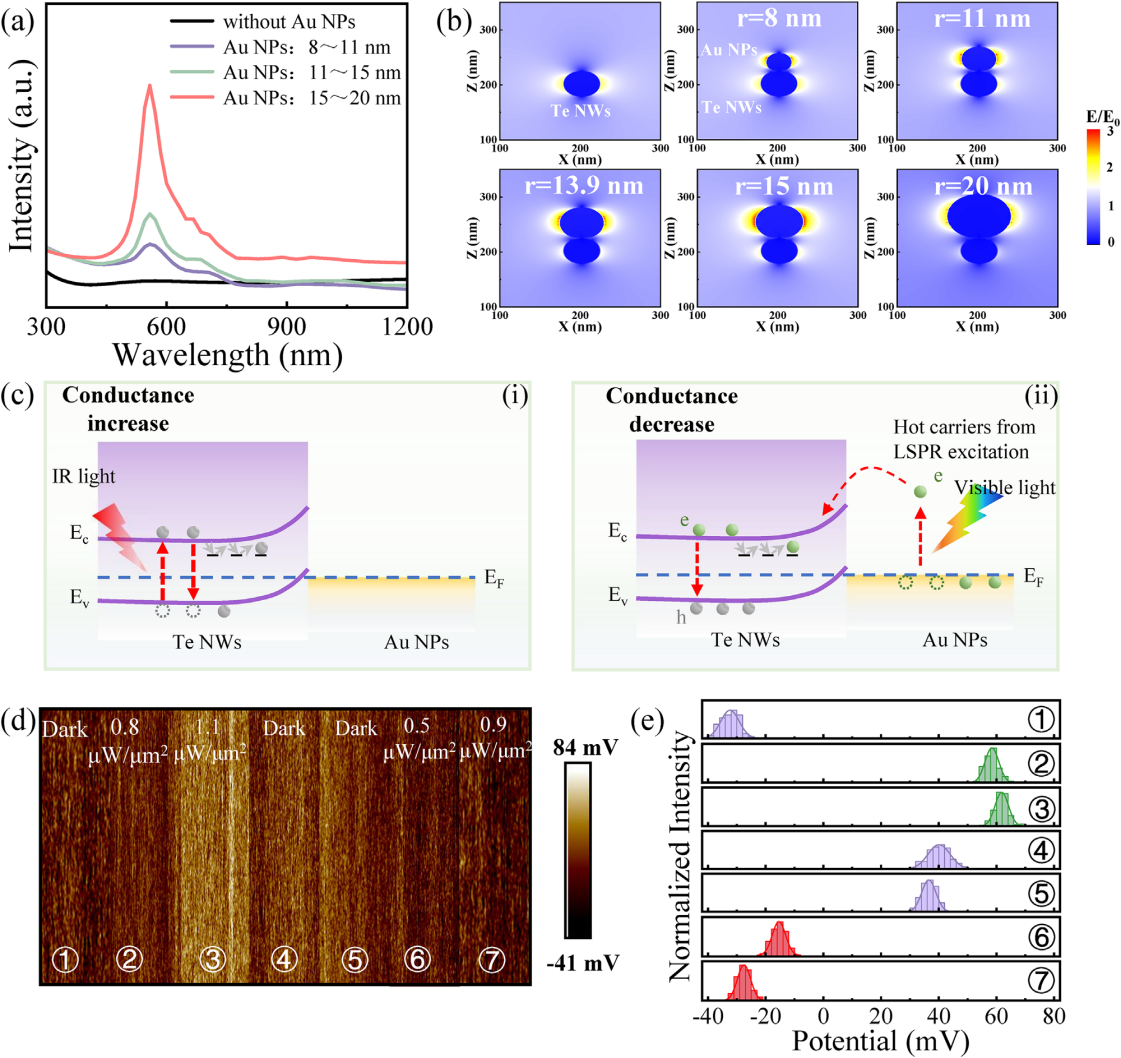
Operating mechanism of the proposed plasmonic optoelectronic memristor. (a) Simulated absorption spectra of the composite materials incorporating Au NPs of various diameters. (b) Evolution of absorption wavelength and of near‐field electromagnetic distribution as a function of Au NPs size. (c) Energy band diagram illustrating the mechanisms of light‐induced charge transfer for bidirectional synaptic modification. (d) Surface potential profiles and (e) the corresponding potential distribution under dark and illumination.

The mechanism of positive photoconductive (PPC) and negative photoconductive (NPC) can be attributed to light‐induced charge transfer and the LSPR effect. Appropriately sized Au NPs enhance the absorbance of fixed‐wavelength visible light in the UV–vis range, exhibiting a pronounced LSPR response. To further investigate this phenomenon, we employed the Finite Difference Time Domain method to simulate the relationship between the Au NPs, absorption wavelength, and corresponding local electric field, as exhibited in Figure 3a,b. The composite films exhibited distinct absorption spectra depending on the size of the Au NPs, which varied with reaction time. The average diameters of the Au NPs at different reaction times are presented in Figure S6. Notably, the LSPR effect is enhanced with increasing Au NP diameter, resulting in substantial local electric field intensification near the Au NPs and Te NWs. Under visible light illumination, collective oscillations of conduction electrons are excited at the Au NP surfaces, generating a localized electromagnetic field that significantly strengthens light‐matter interactions. These oscillations induce intense electric field hotspots, effectively increasing the absorption cross‐section. In the nano‐composite film, the LSPR‐excited hot electrons can be injected into the conduction band of Te NWs, further improving visible‐light absorption. Both experimental and simulation results confirm that the LSPR effect plays a key role in supporting the operating mechanism of the optoelectronic memristor.

A possible mechanism underlying the observed NPC and PPC behaviors is illustrated in Figure 3c. Upon exposure to IR light, electrons in Te NWs are excited from the valence band to the conduction band. The rapid recombination of a portion of these excited electrons with valence band holes gives rise to PPC. The remaining photogenerated electrons become trapped in defect states within the Te NWs. These trapped carriers subsequently undergo transitions between defect states and the conduction band before eventually recombining with holes, resulting in photoconductivity relaxation. In contrast, under 570 nm light illumination, collective oscillations of conduction electrons in Au NPs generate strong nonradiative LSPR. From Figure 3a,b, it can also be observed that the strong LSPR‐induced localized electromagnetic field significantly amplifies the generation of hot carriers in the Au NPs, resulting in hot‐electron injection that dominates over the contribution from the intrinsic excitation of Te. The resulting hot electrons in the Au NPs are excited above the Fermi level and injected into the conduction band of the Te NWs [33, 34]. Given that the Te NWs are p‐type semiconductors, these injected electrons recombine with holes, thereby reducing the intrinsic carrier concentration. As a result, the system exhibits pronounced NPC behavior under 570 nm illumination, as shown in the optoelectronic response in Figure 2b. Further, the spectral dependence of the photoresponse was provided to validate the proposed LSPR mechanism (Figure S7).

To explore the charge carrier redistribution associated with tunable synaptic plasticity, Kelvin probe force microscopy (KPFM) was employed to map the surface potential of the nano‐composite film [35, 36, 37]. When the IR light with the intensity of 0.8µW µm^−2^ was applied, the surface potential increased significantly from its initial value (≈−32 mV) to ≈ 59 mV, as shown in Figure 3d,e. Furthermore, with increasing IR light intensity, the surface potential exhibited a gradual rise and eventually stabilized, indicating a corresponding increase in carrier concentration induced by IR excitation. Conversely, under 570 nm illumination, the surface potential decreased progressively with increasing light intensity. This dynamic and reversible modulation of the surface potential confirms the bidirectional light‐modulated behavior of the device, supporting its synaptic‐like response under different wavelengths.

### 2.4 Demonstration of Motion Detection and Image Recognition

**FIGURE 4:**
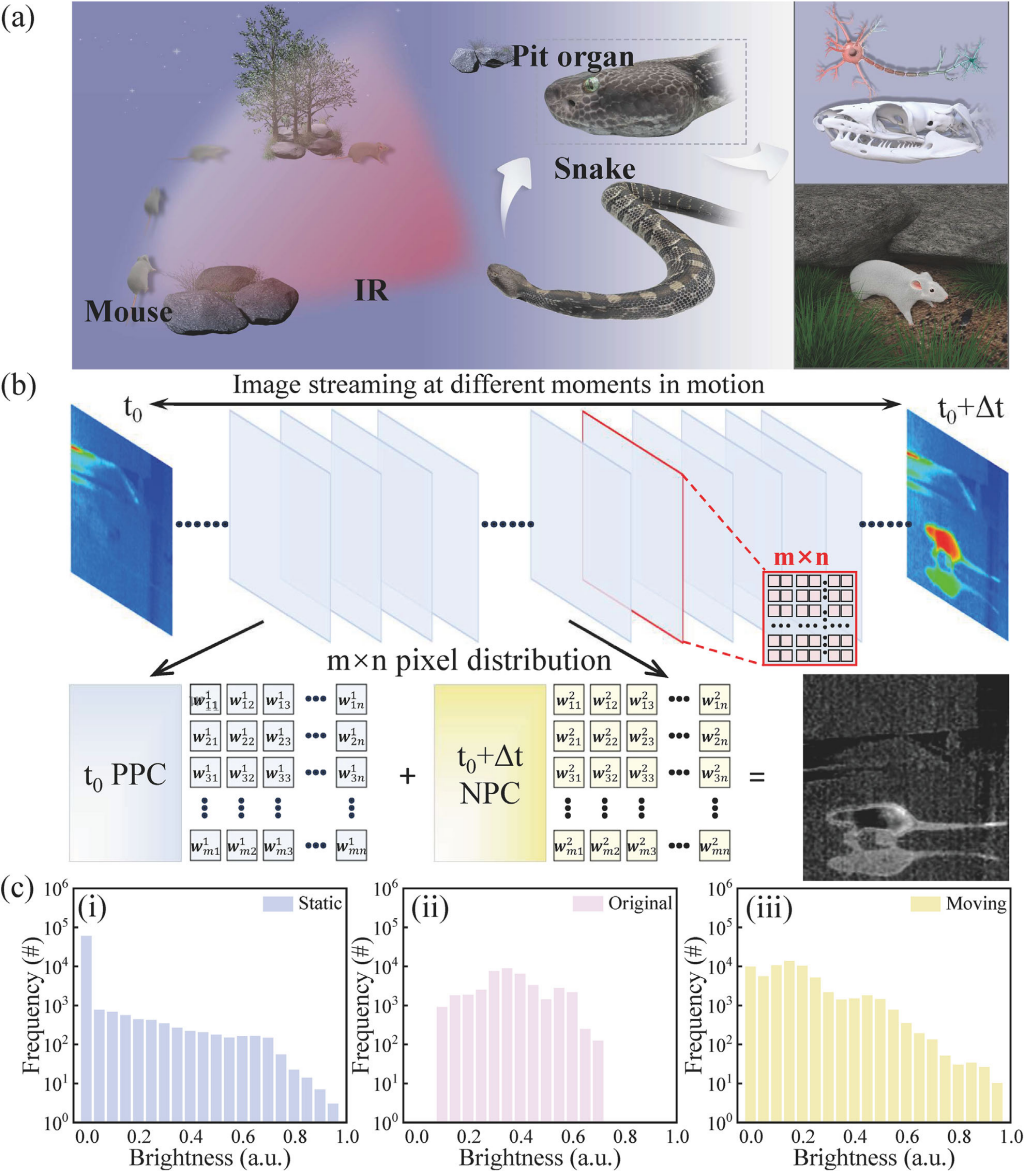
Motion detection enabled by a fully light‐modulated optoelectronic memristor. (a) Schematic illustration of the biological IR sensing mechanism in snakes, which inspired the design of the device. (b) Illustration of motion detection with PPC and NPC in the dark. (c) The brightness distribution of the static image, the original image, and the moving targets image.

Snakes possess a remarkable hyper‐vision capability, as their visual system can detect IR radiation. This mechanism enables them to construct “thermal images” of moving predators or mice [38, 39, 40]. By processing thermal information in their brains, snakes can precisely perceive and track motion in dark environments, such as locating a moving mouse (Figure 4a). Inspired by the infrared sensing mechanism of snakes, the optoelectronic memristor developed in this work can effectively distinguish moving targets from a static background in the dark by leveraging light‐modulated bidirectional synaptic behavior and IR light sensitivity. As illustrated in Figure 4b, the initial scene is an unoccupied grassland, serving as a static background. Within a defined time, a mouse enters the field of view, creating a dynamic contrast relative to the original static environment. As a proof of concept, Figure 4b schematically depicts the working mechanism and operational process of motion target extraction. To implement the detection of a moving target, the dynamic thermal image is segmented into sequential frames, spanning from the initial time t_0_ to the end moment t_0_+Δt. Each thermal image consists of an m×n pixel matrix, where m and n depend on the image resolution. Adjacent frame images can be mapped as PPC and NPC matrices. Adjacent frames are encoded as PPC and NPC matrices. The initial brightness distribution of the thermal image at t_0_, normalized between 0 and 1, is shown in Figure 4b‐ii. To extract moving targets, the m×n PPC and NPC matrices are multiplied with the image brightness data at t_0_ and t_0_+Δt, respectively, enabling a frame‐difference computation [41, 42]. When the scene remains static and there is no moving target, the output pixel values approach zero due to the near‐equal absolute values of the PPC and NPC responses (Figure 4b‐i). The normalized pixel output of 0.25 is selected as the threshold value, in which the pixel output less than 0.25 represents the static state. In contrast, when a mouse appears within a specific time frame, the brightness pixels associated with the moving mouse show values above 0.25, while the corresponding quantitative analysis of conductance change in the pixels matrix is shown in Figure 4b‐iii. The corresponding quantitative analysis of conductance variations is presented in Figure 4b‐iii. These results demonstrate that the proposed optoelectronic memristor successfully enables dynamic detection of moving versus static targets in darkness.

To demonstrate the potential of optoelectronic memristors for neuromorphic computing, the characteristics of the potentiation/depression were investigated. Owing to the inherent mechanical flexibility of the ι‐car organic polymer matrix, the proposed optoelectronic memristor enables the fabrication of flexible synaptic devices suitable for wearable applications. Figure S8a demonstrates the optical pulse sequence applied to the flexible device: 50 consecutive optical spikes at 980 nm followed by another 50 spikes at 570 nm. The corresponding LTP and LTD behaviors were evaluated under three mechanical conditions: flattening, bending, and folding (Figure S8a(i–iii)). A schematic depiction of the device under these mechanical deformations is provided in Figure S9. The observed LTP/LTD characteristics exhibit excellent linearity and symmetry, along with multiple stable conductance states. These features are attributed to the synergistic effects of visible‐light‐induced LSPR and IR‐induced photoconductivity in the nano‐composite film. Further, these behaviors effectively emulate synaptic learning and memory functions, highlighting the memristor's applicability in ANN.

**FIGURE 5:**
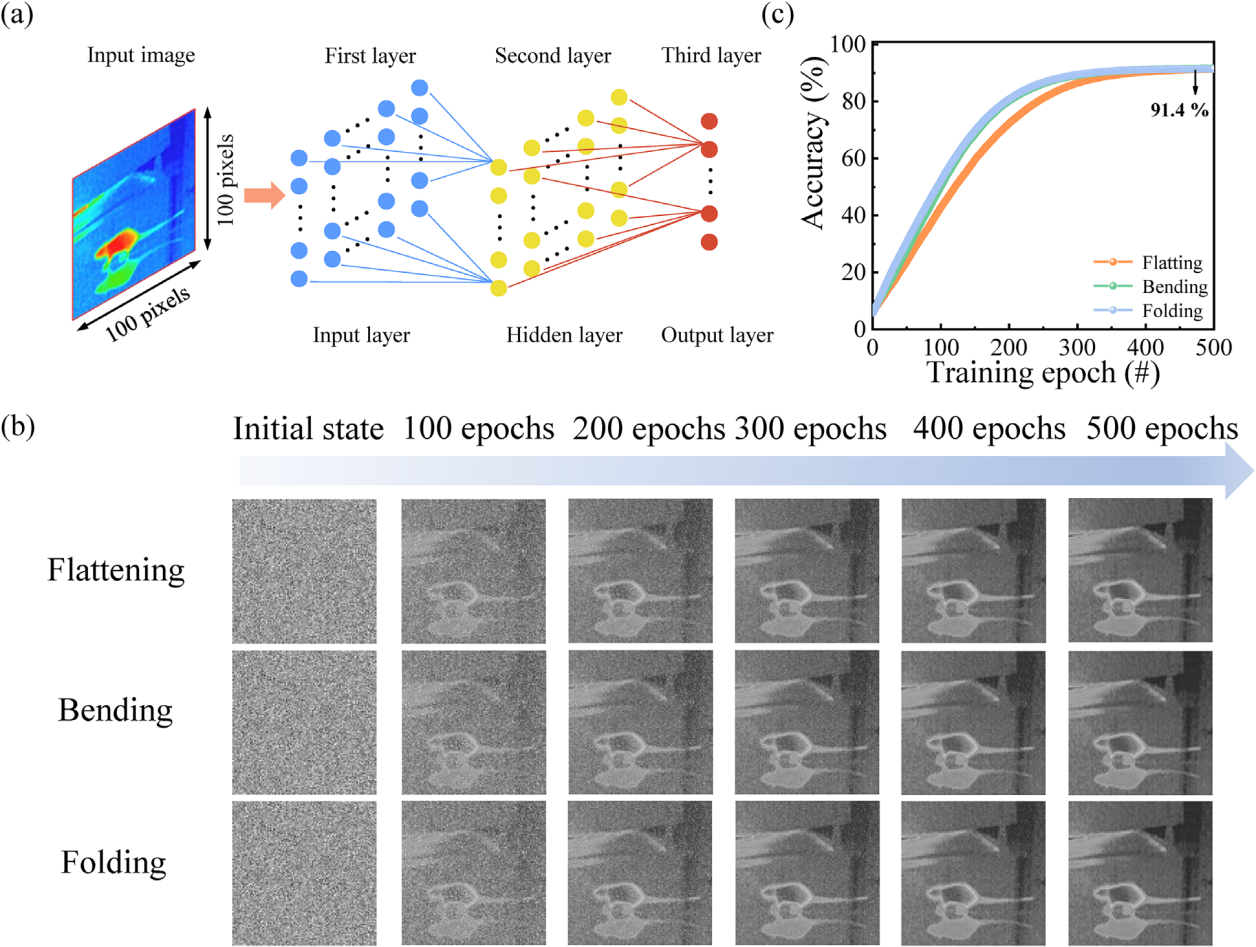
Recognition of the dynamic information using an ANN. (a) Architecture of the ANN incorporating a 100 × 100 memory array. (b) Evolution of images during the learning process based on our plasma optoelectronic memristors under flattening, bending, and folding. (c) Evolution of recognition accuracy as a function of training epochs in the ANNs.

To demonstrate the feasibility of our device for neuromorphic vision applications, a three‐layer ANN was implemented using the proposed optical synapse for training and recognizing dynamic image patterns. As illustrated in Figure 5a, vital information related to moving objects was input into the ANN, leveraging the uniform and stable LTP and LTD behavior of the memristive synapse. For quantitative analysis, the learning accuracy was defined as the similarity between the reconstructed output and the original input image, determined by extracting and identifying dynamic features during the training process. The complete evolution of image states throughout the learning period is provided in Figure 5b. As shown in the simulation results in Figure 5c, the ANN achieved a final recognition accuracy of approximately 91.4% in flattening, bending, and folding states. These results highlight the capability of the memristor‐based system to process dynamic visual information efficiently, demonstrating a promising IR‐sensitive neuromorphic vision platform.

## 3 Conclusion

In summary, we have demonstrated an LSPR all‐optically controlled optoelectronic memristor based on the Te NWs‐Au NPs film nanocomposite structure. By harnessing IR light‐induced PPC and visible light‐induced NPC, reversible, light‐driven synaptic modulation is achieved. Benefiting from the excellent optoelectronic response, various synaptic behaviors, including LTP, PPF, and STDP. Furthermore, optical logic operations were realized through the all‐optical signal synapse in an optical pathway. The fabricated flexible optoelectronic memristor enables excellent consistency of LTP/LTD in flattening, bending, and folding states. Leveraging its bidirectional optical modulation capability, the device also demonstrated robust motion detection in dark environments. Finally, a three‐layer ANN constructed with this synaptic device achieved a recognition accuracy of 91.4% for image inputs, validating its applicability in neuromorphic vision systems. This plasmonic optoelectronic memristor offers a promising platform for integrated sensing, processing, and computing functions in future IR machine vision applications.

## 4 Experimental Section

### 4.1 Synthesis of Te NWs

We synthesized the Te NWs by using a simple hydrothermal method. The poly(vinylpyrrolidone) (1.00 g), Na_2_TeO_3_ (0.922 g), and deionized water (33 mL) were mixed to form a homogeneous solution within 20 min. Then, aqueous ammonia solution (0.8 mL) and hydrazine hydrate (1.4 mL) were added to the homogeneous solution. A 100 mL polytetrafluoroethylene autoclave was employed to hold the above total solution, where the oven temperature is 180°C and the heating time is 3 h. Finally, we removed the reaction solution, which was washed with isopropyl alcohol.

### 4.2 Device Fabrication

Our plasma optoelectronic memristors were fabricated as follows: First, the Te NWs‐Au NPs film was obtained through a chemical transformation reaction. The aqueous solution of Te NWs (5 mL) and HAuCl_4_ solution (1.2 mmol L^−1^) were mixed, where the reaction time was set to 5, 10, and 20 s, respectively. After the reaction, the 0.02 g of ι‐car powder (commercial grade, Sigma–Aldrich, CAS number: 9062‐07‐1) was added to the above three solutions. The Te NWs‐Au NPs/ι‐car film was fabricated using a dip‐coating method at 500 rpm for 5 s and 2000 rpm for 10 s. Finally, the Au planar electrode was deposited by using photolithography and thermal evaporation.

### 4.3 Experimental Measurements

The optoelectrical characteristics of the fabricated memristor were characterized using an integrated measurement platform consisting of a source meter (Keithley 2636B) and a probe station (TTPX, Lake Shore). A xenon lamp with different filters and a 980 nm laser were used as optical excitation sources. All optoelectrical measurements were conducted at room temperature (25°C) under a relative humidity of 30%.

## Author Contributions

Y.T., Z.W., and H.X. conceived the project idea and designed the experiments. J.B. and Y.Z. prepared samples, and performed optoelectrical measurements. J.B., Y.L., X.Z., Z.W., H.X., and Y.L analyzed the data and wrote the manuscript. All authors have given approval to the final version of the manuscript.

## Conflicts of Interest

The authors declare no conflicts of interest.

## Supporting information




**Supporting File**: advs74171‐sup‐0001‐SuppMat.docx.

## Data Availability

The data that support the findings of this study are available from the corresponding author upon reasonable request.
