# Supplementary material for: Infrared Machine Vision System Based on Te NWs‐Au NPs Plasmonic Optoelectronic Memristor for Motion Detection
Source: Adv Sci (Weinh). 2026 Jan 30;13(20):e23162. doi: 10.1002/advs.202523162 (PMC13067755; doi:10.1002/advs.202523162)
Supplement: Supplementary file 1 — Supporting File: advs74171‐sup‐0001‐SuppMat.docx. [file ADVS-13-e23162-s001.docx]

Supporting Information

**Title:Infrared Machine Vision System Based on Te NWs-Au NPs Plasmonic Optoelectronic Memristor for Motion Detection**

*Jingyao Bian^1,2,†^, Yongxing Zhu^1,†^, Ye Tao^1,^*, Zhongqiang Wang^1,^*, Xiaoning Zhao^1^, Ya Lin^1,^*,, Haiyang Xu^1,^*, Yichun Liu^1^*

1. Key Laboratory of Integrated Optoelectronics, Key Laboratory for UV Light-Emitting Materials and Technology (Northeast Normal University), Ministry of Education, 5268 Renmin Street, Changchun 130024, P. R. China

2. School of Science, Heilongjiang University of Science and Technology, Harbin 150020, China

E-mail: taoy506@nenu.edu.cn, [wangzq752@nenu.edu.cn](mailto:wangzq752@nenu.edu.cn), liny474@nenu.edu.cn and [hyxu@nenu.edu.cn](mailto:hyxu@nenu.edu.cn)


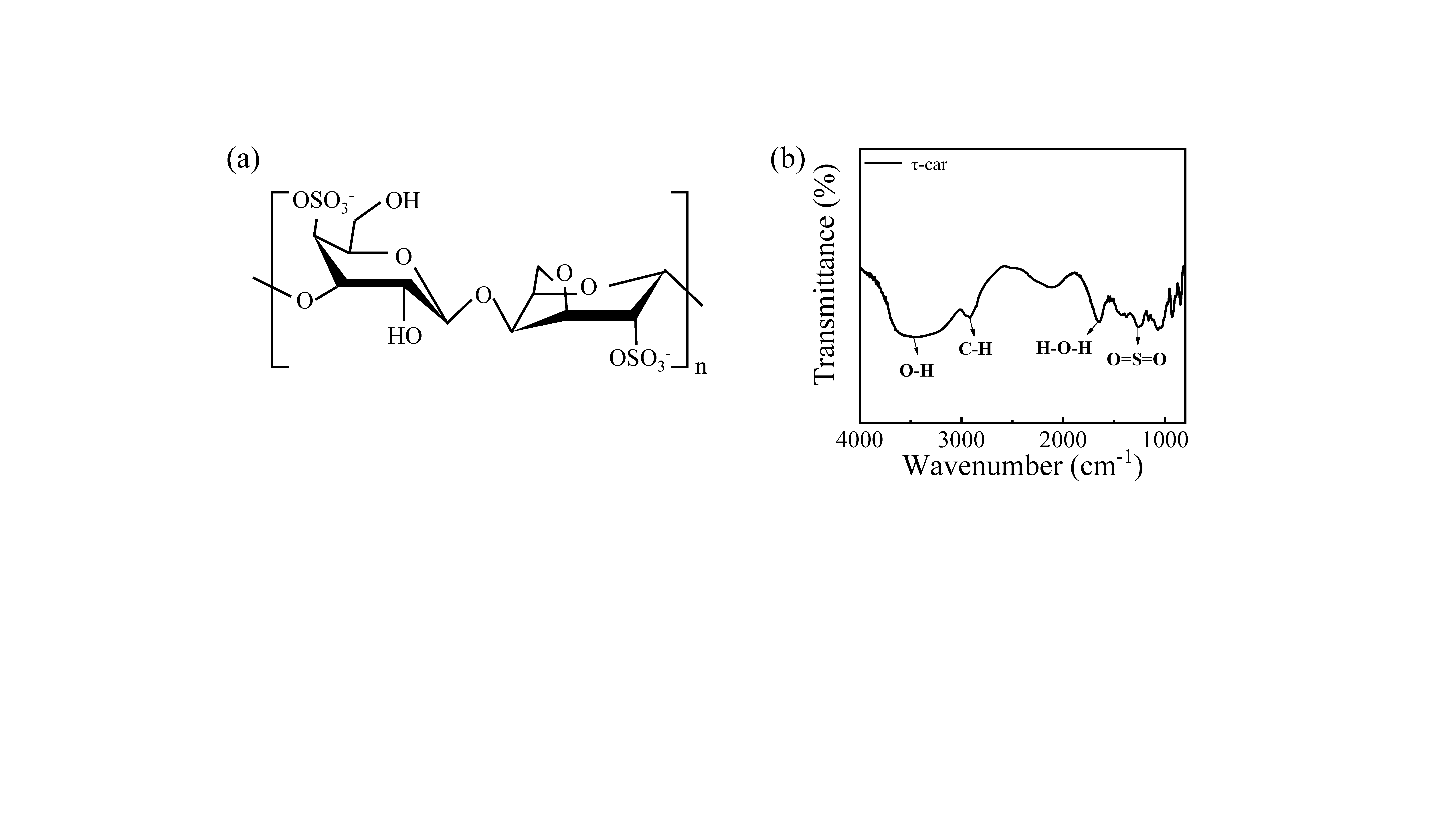


Figure S1. (a-b) The molecule structure and Fourier Transform Infrared Spectrometer of ι-car.


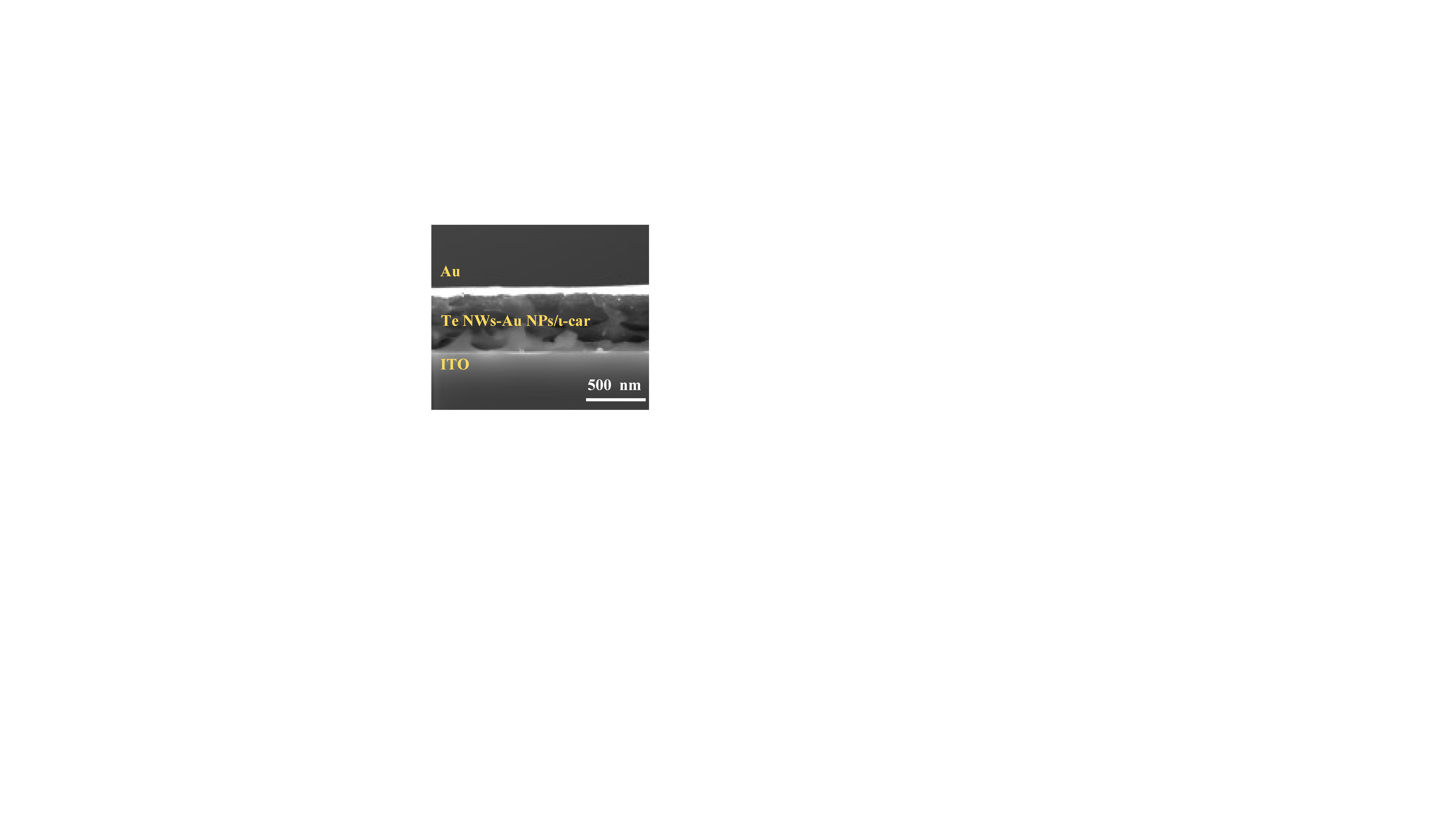


Figure S2. The cross-sectional scanning electron microscope image of optoelectronic memristor.


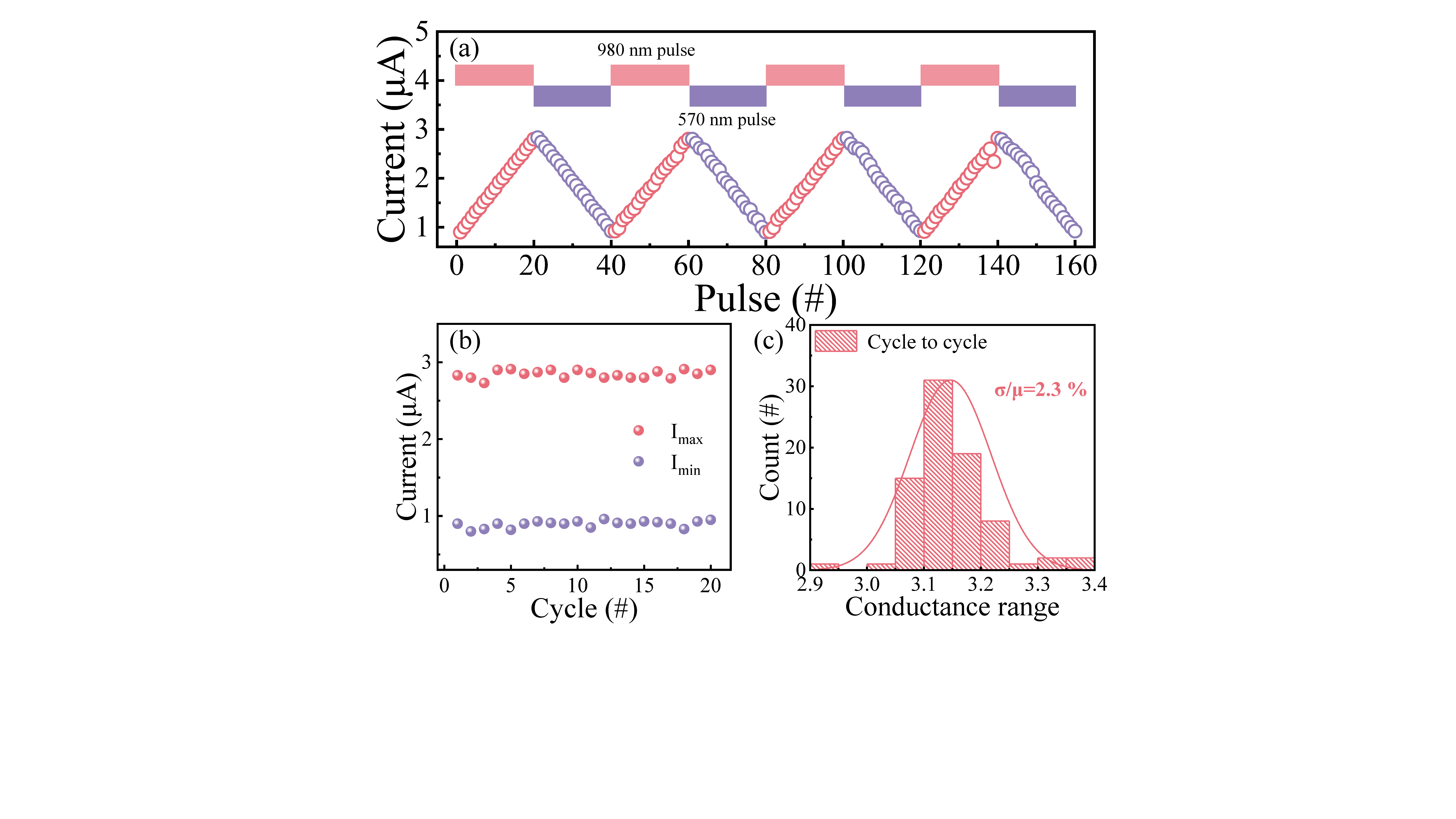


Figure S3. (a) The potentiation and depression behaviors obtained by continuous IR and visible pulses, respectively. (b) Cyclical measurement of the potentiation and depression behaviors. (c) The statistical distribution of conductance range over 80 cycles, and the fluctuation of conductance range can be determined as 2.3 %.


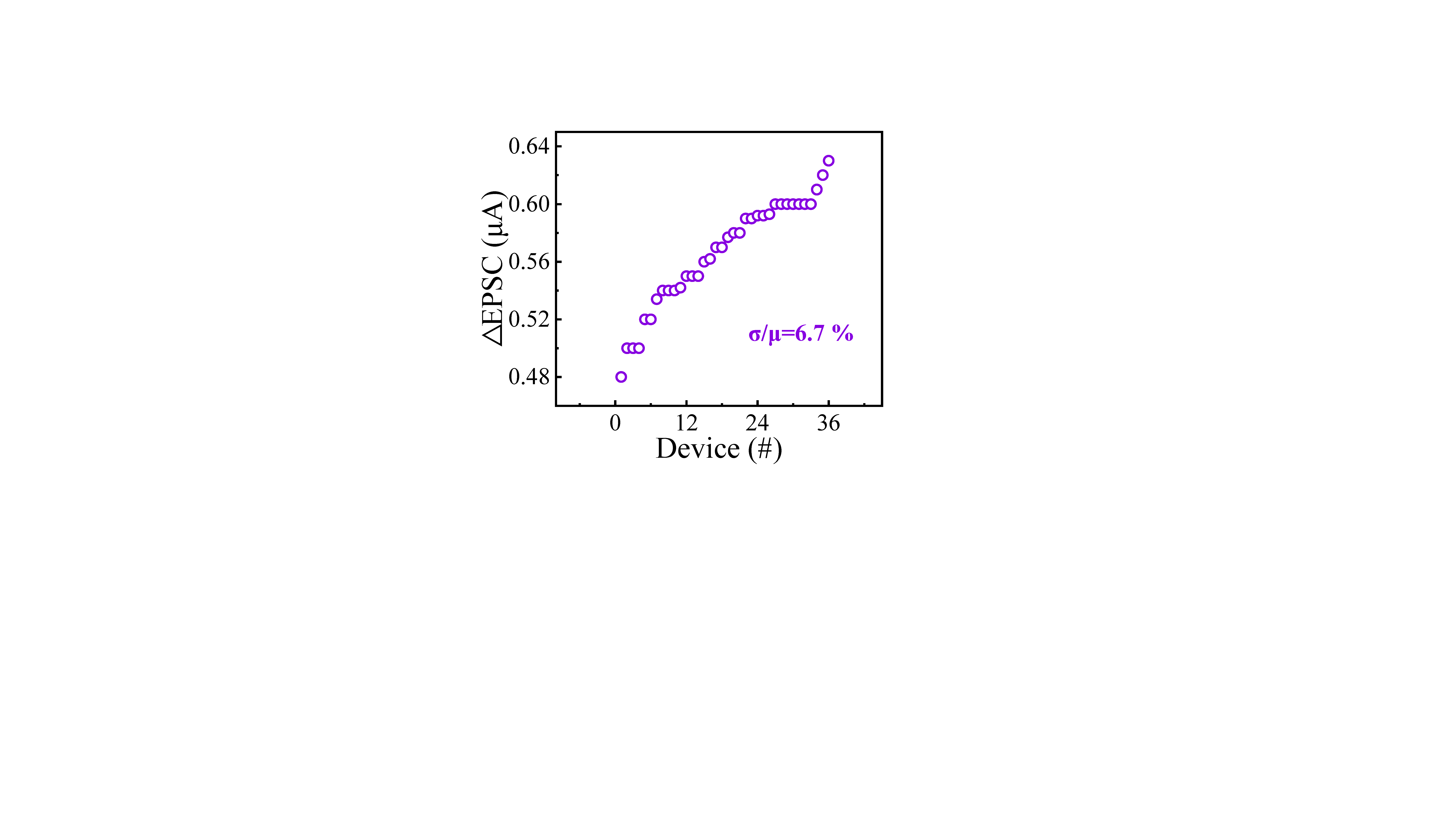


Figure S4. ΔEPSC values induced by optical stimuli, collected across 36 independent memristor devices.


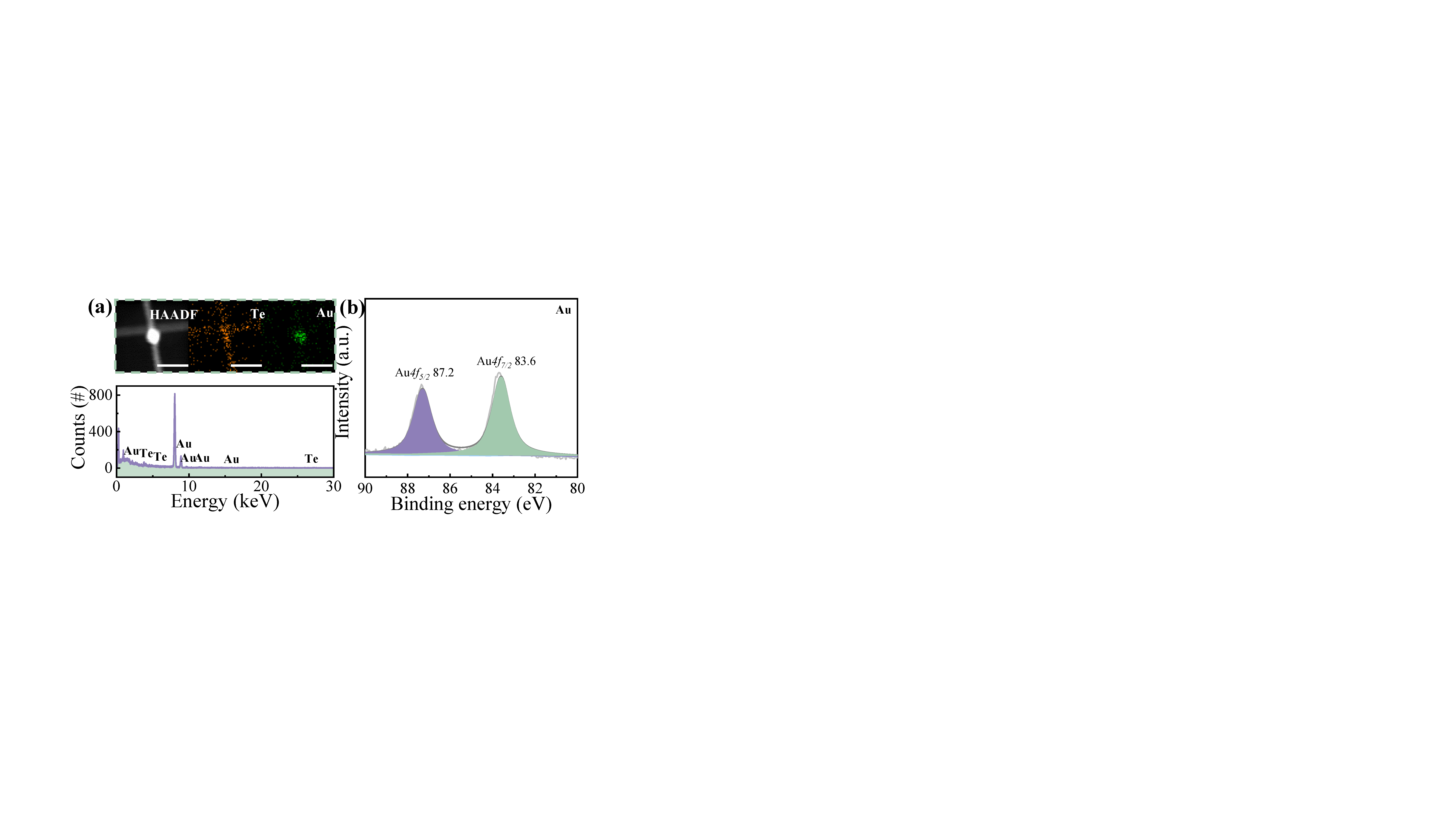


Figure S5. (a) Elemental mapping images and energy dispersive X-ray (EDX) analysis of the Te NWs-Au NPs film. (b) X-ray photoelectron spectroscopy (XPS) core-level spectra of Au 4f states.


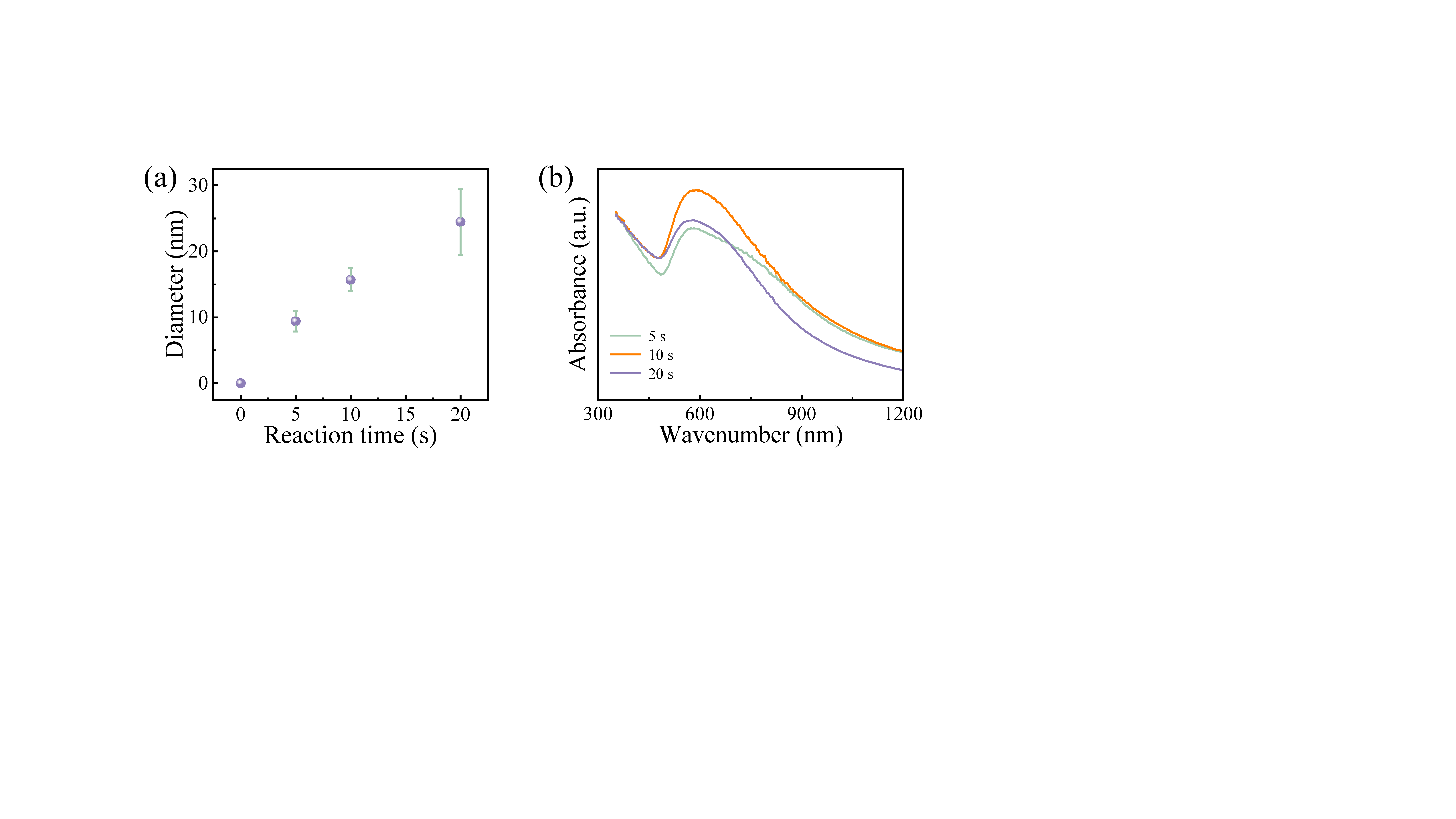


Figure S6. (a) Diameter of Au NPs under different reaction times. (b) The Absorption spectrum of Te NWs-Au NPs heterojunction films with different reaction times.


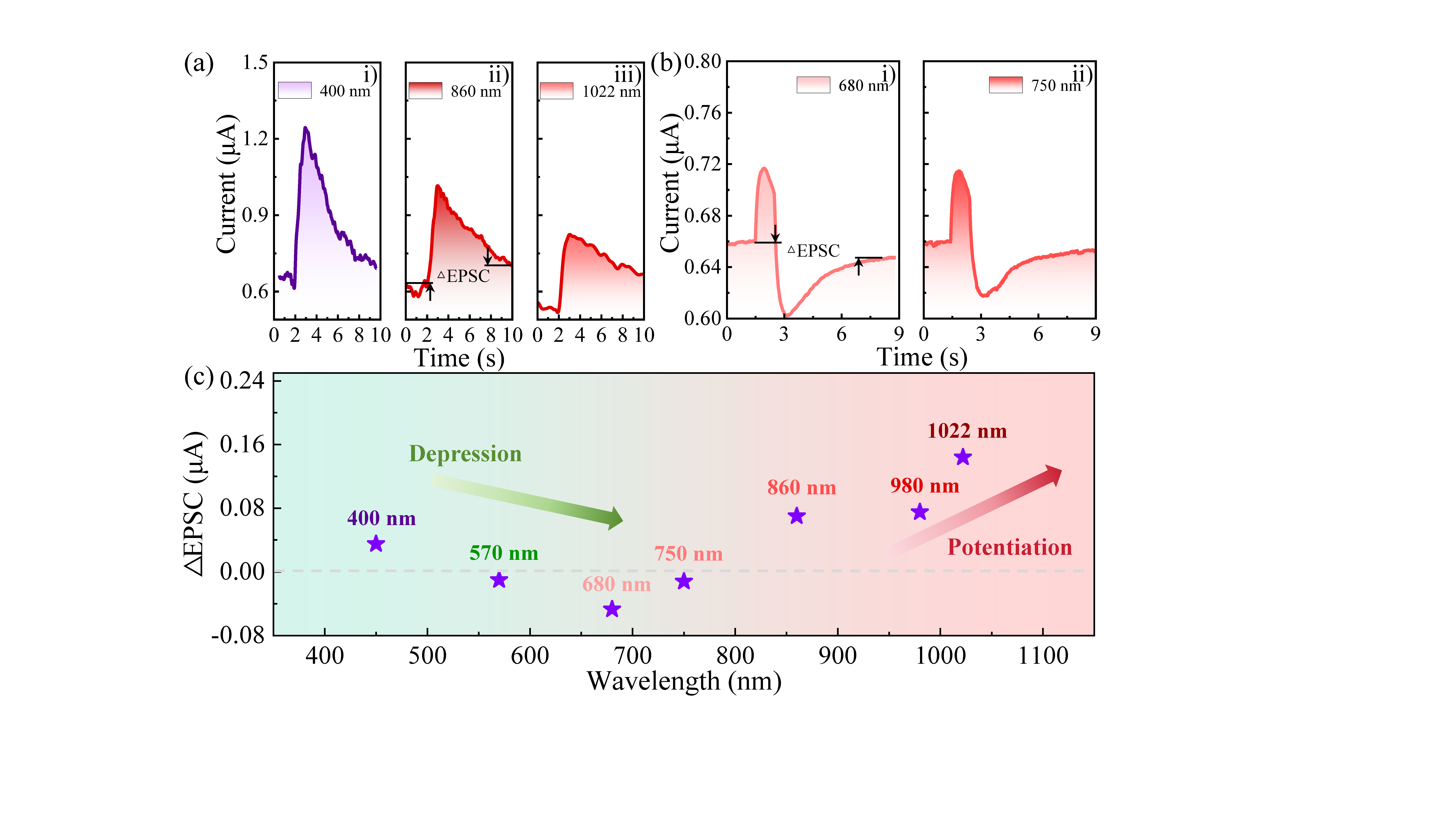


Figure S7. The response current of the device under multiple monochromatic light sources with different wavelengths: (a) 400, 860, and 1022 nm, (b) 680 and 750 nm. (c) Schematic illustration of the wavelength-dependent $\triangle$EPSC response.


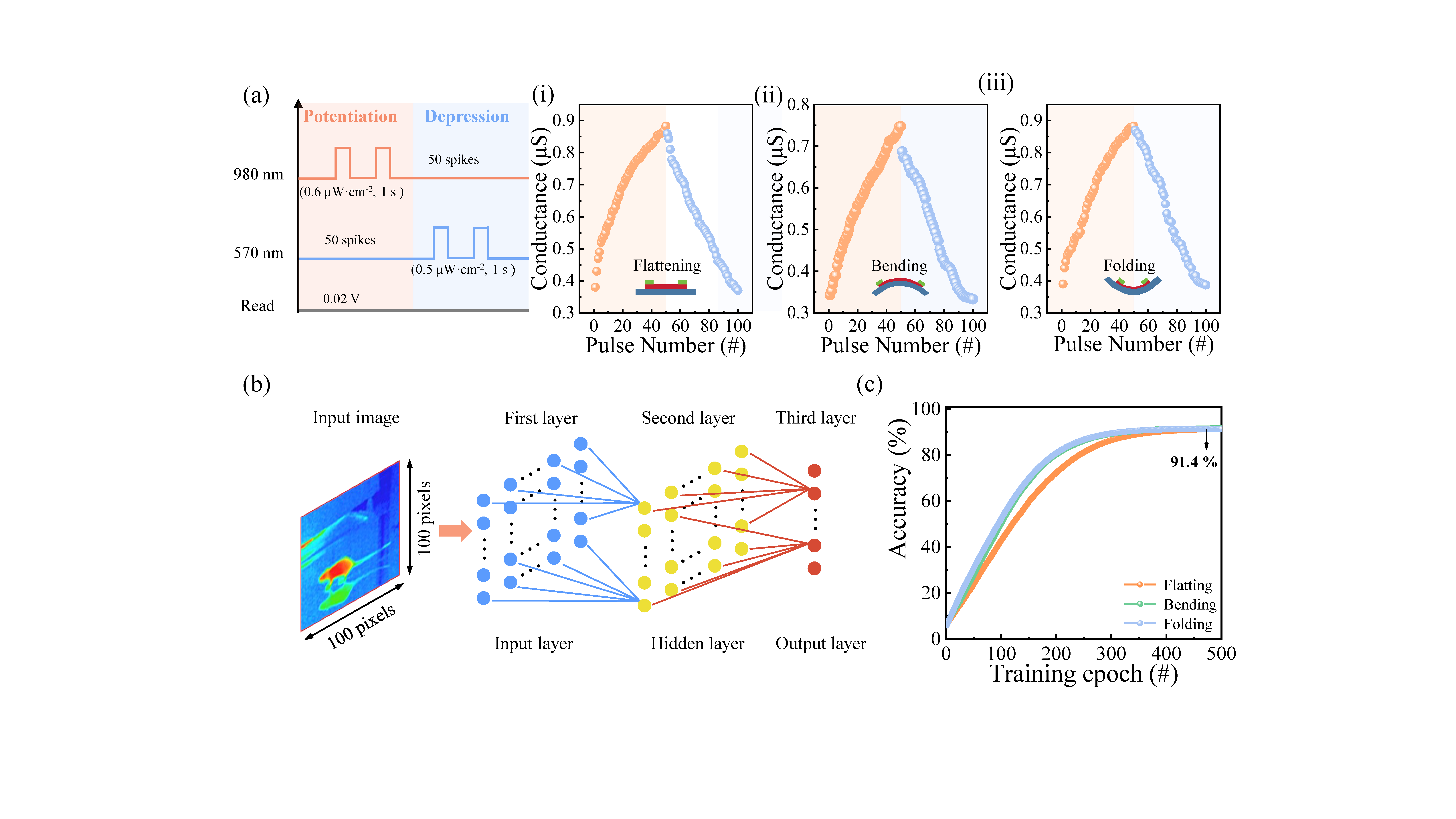


Figure S8. (a) Schematic illustrations of optical spike design for potentiation and depression. (i-iii) Consistent LTP/LTD behaviors of the optical synapse under different mechanism states: flattening, bending, and folding.


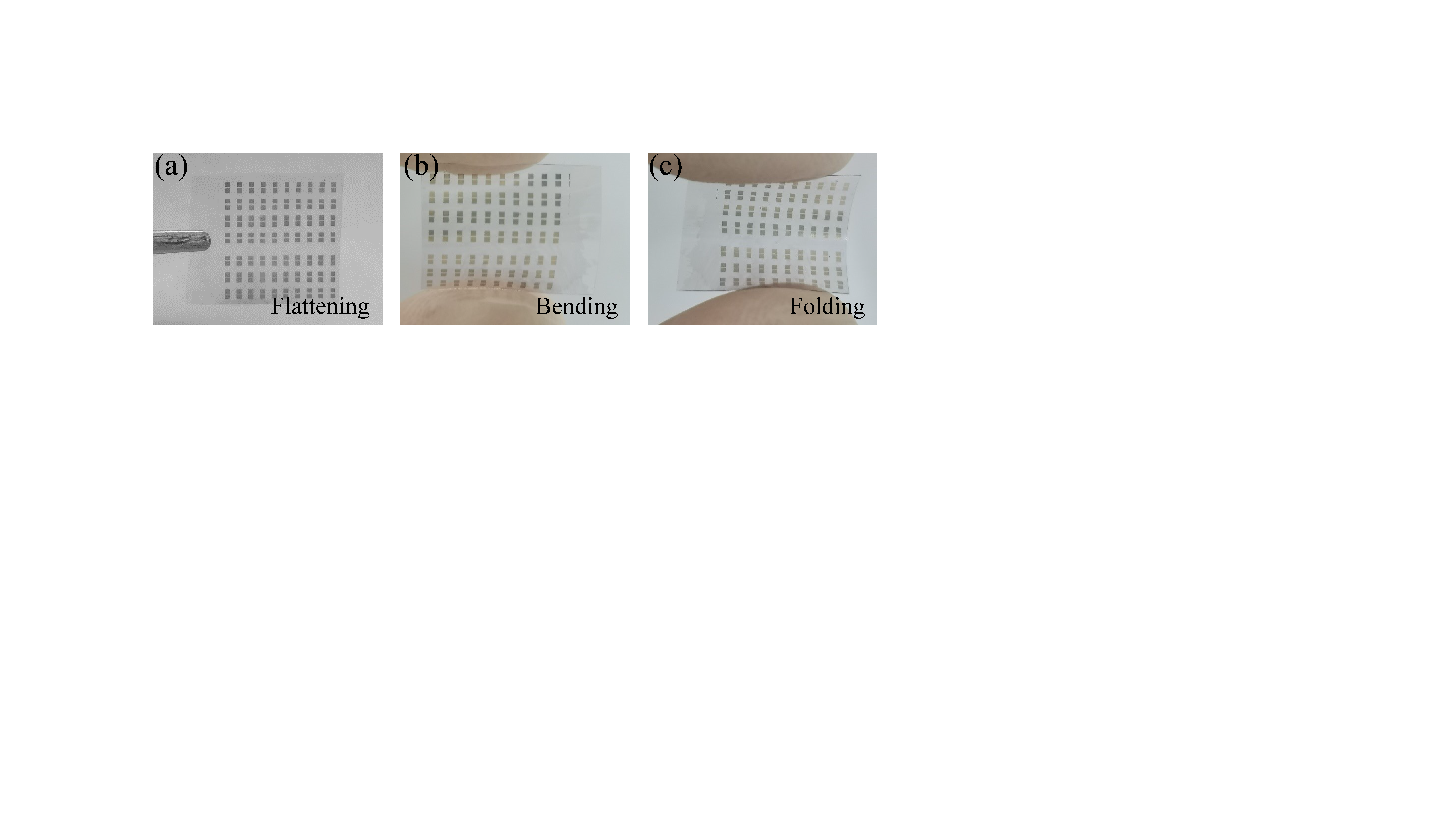


Figure S9. (a-c) Schematic diagram of the device in flattening, bending, and folding state.

Note 1:
The PPF or PPD index decayed with the Δt value. This index is defined as follows:

$$PPF or PPD index=A_{1}e^{{-\triangle t}/{\tau_{1}}}+A_{2}e^{{-\triangle t}/{\tau_{2}}}+C$$

here, Δt is the interval between consecutive light pulses, A₁ and A₂ define the total current variation amplitude, and τ₁ and τ₂ denote the fast and slow decay time constants of the exponential function, respectively.”
